# New Jersey Medical Institute, at Burlington, N. J.

**Published:** 1853-03

**Authors:** 


*New Jersey Medical Institute, at Burlington, N. J.*—A faculty has been organized at Burlington, N. J., with a view to a systematic course of medical instruction during the summer months. The plan embraces familiar didactic, and clinical lectures, but not the conferring of degrees. Dr. Joseph Parrish, editor of the Reporter, will instruct in the theory and practice of medicine. The other departments of medical science are assigned to competent teachers.

